# YB-1 transferred by gastric cancer exosomes promotes angiogenesis via enhancing the expression of angiogenic factors in vascular endothelial cells

**DOI:** 10.1186/s12885-020-07509-6

**Published:** 2020-10-14

**Authors:** Xiaoxia Xue, Jin Huang, Kai Yu, Xinyue Chen, Yini He, Dianjun Qi, Ying Wu

**Affiliations:** 1 Science Experiment Center, China Medical University, 77 Puhe Road, Shenyang North New Area, Liaoning 110122 Shenyang, China; 2 Department of Radiation Therapy, The First Hospital, China Medical University, 518 Chuangxin Road, Hunnan District, Shenyang, 110167 Liaoning China; 3 Department of General Practice, The First Hospital, China Medical University, 155 South Nanjing Street, Heping District, Shenyang, 110001 Liaoning China

**Keywords:** YB-1, Exosome, Gastric cancer, Angiogenesis, IL-8, VEGF, MMP-9, Ang-1

## Abstract

**Background:**

Angiogenesis is important for the progression of gastric cancer (GC). Y-box binding protein 1 (YB-1) predicts advanced disease and indicates neovasculature formation in GC tissues, while the related mechanisms remain elusive. Exosomes mediate intercellular communications via transferring various molecules including proteins, lipids, mRNAs, and microRNAs, while the cargos of GC exosomes and the related mechanisms in GC angiogenesis were rarely reported except for several microRNAs.

**Methods:**

In this study, human umbilical vein endothelial cells (HUVECs) were, respectively, treated by the exosomes isolated from the YB-1 transfected and the control SGC-7901 cells (SGC-7901-OE-Exo and SGC-7901-NC-Exo), and their apoptosis, proliferation, migration, invasion, and angiogenesis were, sequentially, compared. The levels of angiogenic factors including VEGF, Ang-1, MMP-9 and IL-8 in the exosome-treated HUVECs and the GC-derived exosomes were, separately, detected using PCR and Western blotting as well as RNA sequencing assays.

**Results:**

We observed the consistent level of YB-1 in the exosomes and their originated GC cells, and the internalization of exosomes into HUVECs. Comparing with SGC-7901-NC-Exo, SGC-7901-OE-Exo significantly inhibited the apoptosis but promoted the proliferation, migration, invasion, and angiogenesis of HUVECs, within which the increased mRNA and protein levels of VEGF, Ang-1, MMP-9 and IL-8 were demonstrated. Meanwhile, mRNA levels of VEGF, Ang-1, MMP-9 and IL-8 showed no significant difference between SGC-7901-NC-Exo and SGC-7901-OE-Exo, although statistically higher mRNA of YB-1 was detected in the SGC-7901-OE-Exo.

**Conclusions:**

Our findings illustrate YB-1 as the key component of exosome to promote GC angiogenesis by upregulating specific angiogenic factors in the exosome-treated endothelial cells but not in the exosomes themselves.

## Background

Gastric cancer (GC) is the third leading cause of cancer death around the world [1]. Most GC patients were diagnosed at late stage and showed unsatisfied response to traditional therapies, such as chemotherapy and radiotherapy [2, 3]. Angiogenesis is the necessary step for tumor progression; therefore antiangiogenic therapy has earned much attention [3–5]. However, GC patients seldomly benefit from existing antiangiogenesis agents, such as Apatinib and Ramucirumab [3, 6]. Thus, further explorations for the neovasculature mechanisms and novel potential treatment targets in GC are warranted.

In the past decade, studies for angiogenesis have focused on the intercellular communications, with exosomes as the important mediators [7, 8]. Exosomes are nanoscale extracellular vesicles that could be secreted by almost all types of cells and contain variety of biomolecules, such as mRNA and proteins [8, 9]. According to the in-vitro and in-vivo studies, exosomes derived from cancer cells are supposed to mediate vascular formations [7, 8]. Furthermore, exosomes were demonstrated to transfer angiogenic stimulatory molecules to neovasculature related cells, or involve in the reprogramming and modulation of endothelial cells by inducing the expression of pro-angiogenesis genes [10, 11]. In GC, most studies focused on the roles of exosomes in tumorigenesis, metastasis, immune evasion and drug resistance, while the knowledge of exosome angiogenesis is still at its early stage with only several miRNAs reported and deserves further elucidations [2]. Interestingly, Y-box binding protein-1 (YB-1) has been reported the important component of inactive messenger RNA particles (mRNPs), which are also the form of stabilized mRNAs transferred by exosomes during cellular communications [12]. Nontheless, the existence of YB-1 in exosomes and the roles for cancer angiogenesis are still uncovered.

YB-1 is a transcription and translation factor with multiple functions and has been regarded as the potential biomarker as well as therapeutic target in various cancers [13, 14]. YB-1 was originally found to modulate the oncogenesis, cell survival, DNA replication/repair, and drug resistance in cancer cells, and later reported to promote the survival of oxidant-enriched tumorigenic endothelial cells via involving in multiple angiogenic pathways [15]. With mice model of pancreatic and colorectal cancer, YB-1 in endothelial cells was further demonstrated to upregulate the motility of endothelial cells and the secretion of angiogenic factors in extracellular vesicles including exosomes, therefore play an essential role for the tumor formation and vascularization induced by endothelial-cells [16]. In GC, YB-1 was revealed to be the potential prognostic biomarker for GC patients, and the potential indicator for GC angiogenesis in GC tissue specimens [17, 18]. However, the mechanisms of YB-1 for the communication of cancer and endothelial cells and the angiogenesis of cancers are awaiting more detailed declarations.

Currently, we revealed that exosomes derived from YB-1 transfected GC cells (SGC-7901 LV-YBX1) could establish the premetastatic niche by inhibiting the apoptosis and promoting the motility of human umbilical vein endothelial cells (HUVECs), with increased mRNAs and proteins of intracellular VEGF, Ang-1, MMP-9 and IL-8 observed. Nontheless, genomic sequencing showed the almost equal mRNAs of the four angiogenic factors in the exosomes derived from SGC-7901 LV-YBX1 and the control cells (SGC-7901 LV-NC). With our study, we disclosed the novel mechanisms of exosome-induced angiogenesis in GC and the potential functions of YB-1 in the extracellular exosomes.

## Methods

### Cell lines and cell culture

The GC cell line SGC-7901, HGC-27, MKN45, KATOIII, BGC-823, NUGC-4 were purchased from American Type Culture Collection (ATCC,USA), and cultured in RPMI-1640 medium (HyClone, Thermo Scientific, USA) supplemented with 10% fetal bovine serum (FBS, HyClone, Thermo Scientific, USA) and 1% penicillin/streptomycin (HyClone, Thermo Scientific, USA). HUVECs were purchased from the Sciencell and cultured in ECM consisting of 500 ml of basal medium, 25 ml of fetal bovine serum, 5 ml of endothelial cell growth supplement (ECGS) and 5 ml of penicillin/ streptomycin solution (P/S). SGC-7901 LV-YBX1 and LV-NC Cells were cultured in RPMI-1640 medium (HyClone, Thermo Scientific, USA) supplemented with 10% fetal bovine serum (FBS, HyClone, Thermo Scientific, USA) and 1% penicillin/streptomycin (HyClone, Thermo Scientific, USA). All cells were incubated in a standard humidified incubator in 5% CO_2_ at 37 °C.

### Construction and infection of recombinant lentiviral vectors of YB-1

We designed YB-1 and negative control lentiviral vector based on the gene sequence of human YBX1 (NM_004559; Forward: GGGGACAAGAAGGTCATCGC; Reverse: CGAAGGTACTTCCTGGGGTTA). By using calcium phosphate transfection kit (Invitrogen, USA), the lentivirus was generated by co-transfection of 293 packaging cells with the modified pGV358-EGFP viral vector and the pHelper 1.0 and pHelper 2.0 helper plasmids (GeneChem Technology, Shanghai, China). The YB-1 overexpression lentiviral vector was named LV-YBX1(LVKL15852–21), and the negative control lentiviral vector was named LV-NC(KL8781–1). SGC-7901 cells were infected with LV-YBX1 or LV-NC at MOI = 10 in 5 μg/ml polybrene (infection enhancer) for 16 h when the medium was refreshed. After 72 h, 4 μg/ml of Puromycin (Sigma Aldrich, GER) was supplemented into the transfection system for 48 h. To identify the successful establishment of SGC-7901 LV-YBX1 and LV-NC cell lines, the fluorescence of cells was measured using the fluorescence microscope, and the infection efficiency calculated by the ratio of fluorescent cells to all cells should be higher than 75%.

### Exosome isolation

The GC cell line HGC-27, MKN45, KATOIII, BGC-823, NUGC-4, SGC-7901, and SGC-7901 LV-YBX1 and LV-NC cells were cultured until 80% confluent, then collected the cell culture medium after 48–72 h. Exosomes were isolated from the medium by differential centrifugation according to previous publications [19]. The cell culture medium was centrifuged at 300 g for 10 min and 3000 g for 20 min at 4 °C to remove the cells and other debris, 10,000 g for 30 min at 4 °C to further remove other vesicles. The supernatant was pelleted at 100000 g for 70 min at 4 °C by ultracentrifugation (Kendro, USA); exosomes were collected and resuspended in PBS. The total exosome protein concentration was measured by the Enhanced BCA Protein Assay Kit (Beyotime, China). The exosomes derived from SGC-7901 LV-YBX1 and LV-NC cells were, respectively, named as SGC-7901-OE-Exo and SGC-7901-NC-Exo.

### Exosome identification by electron microscopy

Exosomes were fixed in 1% glutaraldehyde. Dropping approximate 10 μl of suspension in a formvar-carbon copper electron microscopy grids then stained with 2% phosphotungstic acid and obtained images using a digital camera H-7650 (Hitachi, JAN).

### Exosome labeling

According to the manufacturer’s recommendations, exosomes were labeled with PKH26 Red Fluorescent Cell Linker Kit (Sigma-Aldrich, GER) for General Cell Membrane Labelling. Briefly, exosome pellets were resuspended in 1 ml Diluent C, and then incubated for 5 minutes with the stain solution prepared by mixing 4 μl PKH26 with 1 ml Diluent C. To stop the staining, an equal volume (2 ml) of serum was added and incubated with the staining system for 1 min. Labeled exosomes were washed more than twice with 10 ml of complete medium (400 g for 5 min).

### Confocal microscopy

HUVECs on the coverslips inside a petri dish filled with the appropriate culture medium were, respectively, incubated with the labeled SGC-7901-OE-Exo, SGC-7901-NC-Exo, and PBS for 12 h at 37 °C. Then, the medium in the dishes was replaced by the prewarmed (37 °C) PBS containing CFDA-SE (0.5–25 μM; Vybrant® CFDA SE Cell Tracer Kit; Invitrogen, USA) to label the cells for 15 min at 37 °C. After that, HUVECs were incubated with fresh and prewarmed culture medium for 30 min at 37 °C, and then fixed using 4% formaldehyde for 15 min at room temperature, and stained nucleus using DAPI (Beyotime, China) for 5 min. Finally, cells were observed and analyzed with a laser scanning confocal microscope (Olympus FV1000, Tokyo, JAN).

### Cell proliferation assay

HUVECs were inoculated in a 96-well plate (2 × 10^3^ cells in 200 μl of culture media per well) and cocultured, respectively, with SGC-7901-OE-Exo (50, 100, 200, and 400 μg/ml), SGC-7901-NC-Exo (400 μg/ml), and PBS in an incubator containing 5% CO_2_ at 37 °C for 1–3 days. The cell proliferation at 24 h, 48 h and 72 h was, respectively, measured by 3-(4,5-dimethylthiazol-2-yl)-2,5-diphenyltetrazolium bromide (MTT) assay. After 4 h incubation of HUVECs with MTT (Sigma-Aldrich) stock solution (5 mg/ml, 20 μl for each well), 200 μl of dimethyl sulfoxide (DMSO, Sigma-Aldrich, GER) was added to each well for 10 min, then the absorbance was measured at 570 nm using a Multimode Plate Readers (Molecular Decice, USA). OD values were calculated from 3 independent experiments.

### Flow cytometry assays for apoptosis evaluation

Annexin V-FITC Apoptosis Detection Kit (kegen biotech, China) was used to evaluate the percentage of apoptosis cells. HUVECs were inoculated in a 6-well plate and cocultured, respectively, with SGC-7901-OE-Exo (400 μg/ml), SGC-7901-NC-Exo (400 μg/ml), and PBS for 24 h at 37 °C in 5% CO_2_. The cells were collected and washed with PBS twice (2000 rpm for 5 min) and resuspended in 500 μl of binding buffer to adjust the density of cell suspension as 1–5 × 10^5^ /ml. Then, a volume of 5 μl Annexin V-FITC and 5 μl propidium iodide (PI) were gently mixed with cell suspensions. After incubation at room temperature in the dark for 10 min, cell apoptosis was detected by flow cytometer with LSRFortessa (BD Biosciences, San Diego, USA), and the mean of apoptosis index (%) was analyzed using BD FACSDiva software.

### Scratch wound migration assay

HUVECs were seeded in a 6-well plate (1 × 10^4^ cells/well), and a “scratch” (or a cell-free area) was created with a 200 μl plastic pipette tip in the monolayer (at time 0). Cells were then washed with PBS to remove loose cells and co-cultured, respectively, with SGC-7901-OE-Exo (200 and 400 μg/ml), SGC-7901-NC-Exo (400 μg/ml), and PBS for 6 h and 12 h. The distance between the two sides of the wound was photographed with an Olympus IX51 microscope (Olympus, JAN). Each wound area was measured using Image J image analyzing software and evaluated using the wound area calculated as a percentage of the initial wound area.

### Transwell assays

Invasion assays were performed in 24-well Transwell™ chambers (Costar, USA). The upper and lower culture compartments were separated by polycarbonate filters with 8 μm pore size and the upper chamber was coated with Matrigel matrix (0.8 mg/ml, BD Biosciences, USA) before seeding the cells. Migration assays were performed without Matrigel matrix precoated. With the lower chamber filled with FBS-free medium, HUVECs (1 × 10^5^) were cocultured, respectively, with SGC-7901-OE-Exo (200 and 400 μg/ml), SGC-7901-NC-Exo (400 μg/ml), and PBS in the upper chamber for 24 h at 37 °C in 5% CO_2_. After the non-invading/non-migrating cells were removed using cotton swabs, the invading/migrating cells were fixed and stained with 0.2% crystal violet (Sigma, GER), and then counted with light microscopy (Leica, USA).

### HUVEC tube formation on Matrigel

To determine the influence of exosomes on in vitro vascular tube formation, SGC-7901-OE-Exo (400 μg/ml), SGC-7901-NC-Exo (400 μg/ml), and PBS were applied, respectively, to HUVECs plated on Matrigel in FBS-free endothelial basal medium. Then the cells were incubated for 6 h and calculated by Olympus IX51 microscope (Olympus, JAN).

### Reverse transcription (RT)-PCR and quantitative real-time RT-PCR

HUVECs were cocultured with SGC-7901-OE-Exo (200 and 400 μg/ml), SGC-7901-NC-Exo (400 μg/ml), and PBS for 24 h at 37 °C in 5% CO_2_. Based on the manufacturer’s instruction, total RNA was extracted from HUVECs by TRIzol (Invitrogen, USA) and reversely transcripted to cDNA with the PrimeScript® RT reagent kit (TAKARA, JAN). The amplification of human VEGF, Ang-1, MMP-9 and IL-8 was, respectively, performed with the SYBR Green master mix kit in a LightCycler® 480 Real-Time PCR System (Roche, CH). Target specific PCR conditions were: 30 s at 95 °C, followed by 40 cycles of 5 s at 95 °C, and each one cycle of 20 s at 60 °C, 10 s at 95 °C, 10 s at 60 °C, and 30 s at 40 °C. The ∆∆Ct method was used to calculate the relative expression of target genes by normalizing to GAPDH mRNA level. The sequences of PCR primers in the present study were as follows: VEGF forward sequence 5′-TCACAGGTACAGGGATGAGGACAC-3′ and reverse sequence 5′-CAAAGCACAGCAATGTCCTGAAG-3′; IL-8 forward sequence 5′-CAAACATGATCTGGGTCATCTTCTC-3’and reverse sequence -GCTCGTC GTCGACAACGGCTC-3′; Ang-1 forward sequence 5′-TGATGGACTGGGAAGGGAAC-3’and reverse sequence 5′-AGCGTCCTTTGTGCTGAAAT-3′; MMP-9 forward sequence 5′-TAGGGCTCCCGTCCTGCTT − 3’and reverse sequence 5′-CCACCTCCACTCCTCCCTTTC-3′; GAPDH forward sequence 5′- GGGCTGGCATTGCTCTCA-3’and reverse sequence 5′- TGCTGTAGCCGTATTCATTGT − 3′.

### Western blot analysis

Cells and exosomes were lysed in RIPA lysate (Beyotime Biotechnology, Jiangsu, China), total proteins concentrations were determined using the BCA method (Beyotime Biotechnology, Jiangsu, China). Proteins were separated by SDS-polyacrylamide (10%) electrophoresis, electrotransferred onto PVDF membranes and blocked with 5% BSA in TBS with Tween 20 for 2 h at room temperature. The membranes were, respectively, incubated with primary antibodies YB-1, VEGF, Ang-1, MMP-9, IL-8, HSP-70 and GAPDH (Abcam, USA) overnight at 4 °C, and followed by incubation in the corresponding HRP-conjugated secondary antibodies for 2 h at room temperature. The membranes were visualized using the ECL detection kit (Themo, USA), and protein expressions were detected using Electrophoresis Gel Imaging Analysis System (DNR Bio-Imaging Systems, ISR). Protein expressions were semiquantified by densitometry using the ScanImage software and normalized by GAPDH and HSP-70 levels.

### RNA sequencing assays

A total of 10 ml cell culture media was mixed with RiboTM Exosome Isolation Reagent and exosomes were isolated according to the manufacturer’s instructions (RiboBio, Guangzhou, China). Total RNA of exosomes was sequenced by RiboBio. According to the instructions provided with the NEBNext® Ultra™ RNA Library Prep Kit for Illumina (NEB, USA), RNAs fragmented to approximately 200 bp were subjected to first and second strand cDNA synthesis followed by adaptor ligation and enrichment with a low cycle. The purified library products were evaluated using the Agilent 2200 TapeStation and Qubit®2.0 (Life Technologies, USA) and then sequenced (2 × 150 bp) using a HiSeq30000. Based on raw data, the clean reads were obtained after removal of reads containing adapter, ploy-N and the low-quality ones. Paired-end reads were aligned to the mouse reference genome mm10 with HISAT2. HTSeq was used to count the reads numbers mapped to each gene. Differentially expressed genes were identified according to the criteria of | log 2(fold change) | > 1.5 and adjusted *p*-value < 0.05 by DEseq using read counts as input.

### Statistical analysis

The data values were expressed as mean ± SD of three independent experiments and statistical analysis using SPSS 22.0 software. Differences were examined using Student’s t-test or one-way analysis of variance considered to be statistically significant with a *P* value of 0.05 or less.

## Results

### Identification of YB-1 expression in GC-derived exosomes

**Fig. 1:**
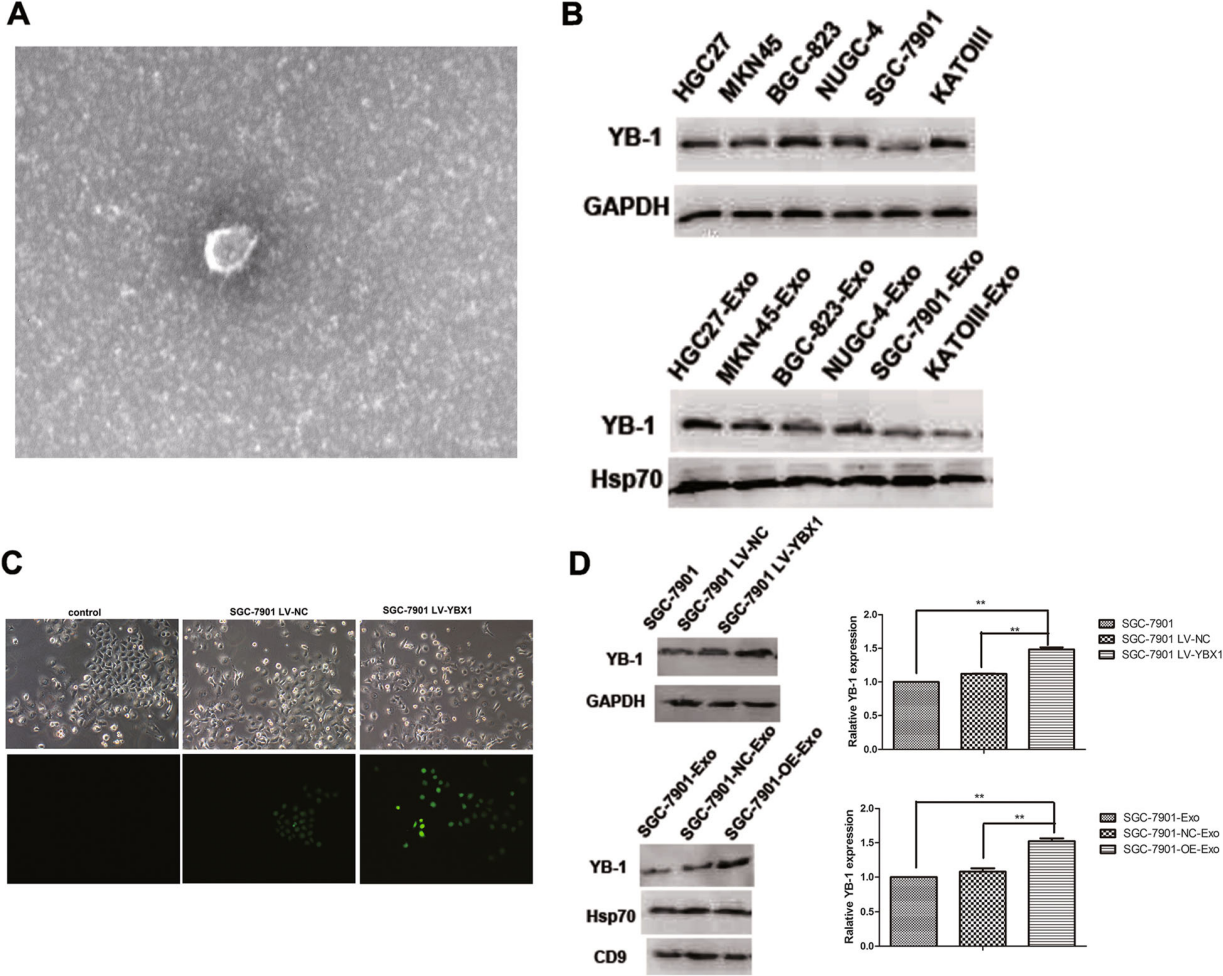
Confirmation of exosome from SGC-7901 cells by differential centrifugation. **a** Transmission electron microscopy (TEM) demonstrates successful isolation of exosome-sized particles from SGC-7901 cell. **b** Western blots of YB-1 in the GC cell lines and the corresponding exosomes respectively. **c** Confocal microscopy images showing YB-1 labeled with green fluorescence in SGC-7901 cell. **d** Western blot and the corresponding quantitative analysis for YB-1, CD9 and Hsp70 in the SGC-7901, SGC-7901-NC and SGC-7901-OE cells and the corresponding exosomes. (*, *P* < 0.05, **, *P* < 0.01)

Based on the observations of electron microscope and western blot assays, it is identified that the exosomes were derived successfully from GC cells according to the consistent shape and size with the previous descriptions (30-110 nm sized vesicles) and the expression of exosome markers including CD9 and Hsp70 (Fig. 1a, b and d). With six GC cell lines, YB-1 was found to express in both GC cells and the corresponding exosomes, and the expression levels of YB-1 in exosomes seem to orchestrate with those in the original GC cells (Fig. 1b). Furthermore, SGC-7901 LV-YBX1 and LV-NC cells were established successfully (Fig. 1c), and that YB-1 expression in the SGC-7901-OE-Exo was confirmed to be higher than in the SGC-7901-NC-Exo, which is coincident with the YB-1 levels in the originated SGC-7901 LV-YBX1 and LV-NC cells (Fig. 1d).

### Internalization of GC exosomes in HUVECs

**Fig. 2:**
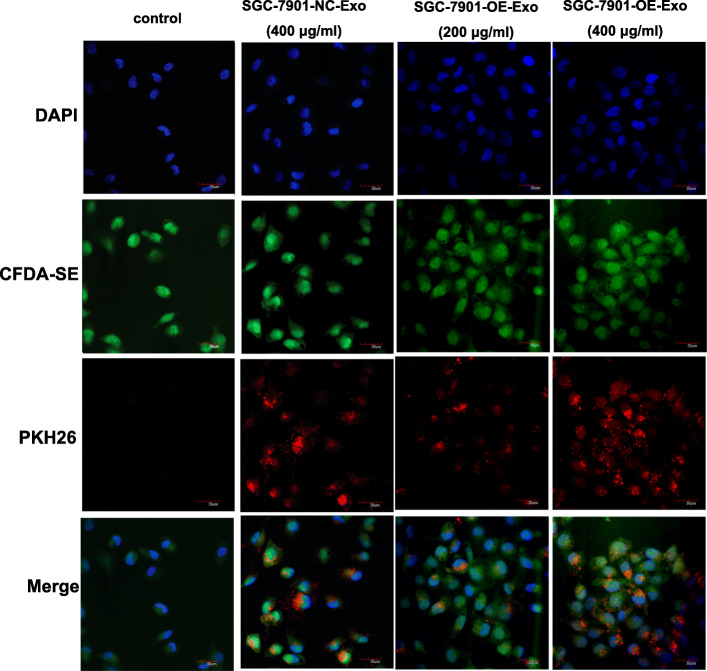
Exosome internalization in HUVECs. As shown in the confocal images, red fluorescent labeled exosomes were taken up by green fluorescent labeled HUVECs after 12 h coculture

Combining fluorescence microscope observation and confocal high-content analysis, the internalization efficacy of GC exosomes in HUVECs was compared using different concentrations of SGC-7901-OE-Exo and SGC-7901-NC-Exo. As shown in Fig. 2, exosomes labeled by PKH26 (red) were efficiently taken up by HUVECs labeled by CFDA-SE (green) after12 h incubation. Moreover, the uptake of exosomes by HUVECs was related with the concentration of exosomes but not the YB-1 levels, and that higher concentration of exosomes seem to induce more internalization of exosomes in HUVECs.

### High YB-1 exosomes irritate proliferation but reduce apoptosis of HUVECs

**Fig. 3:**
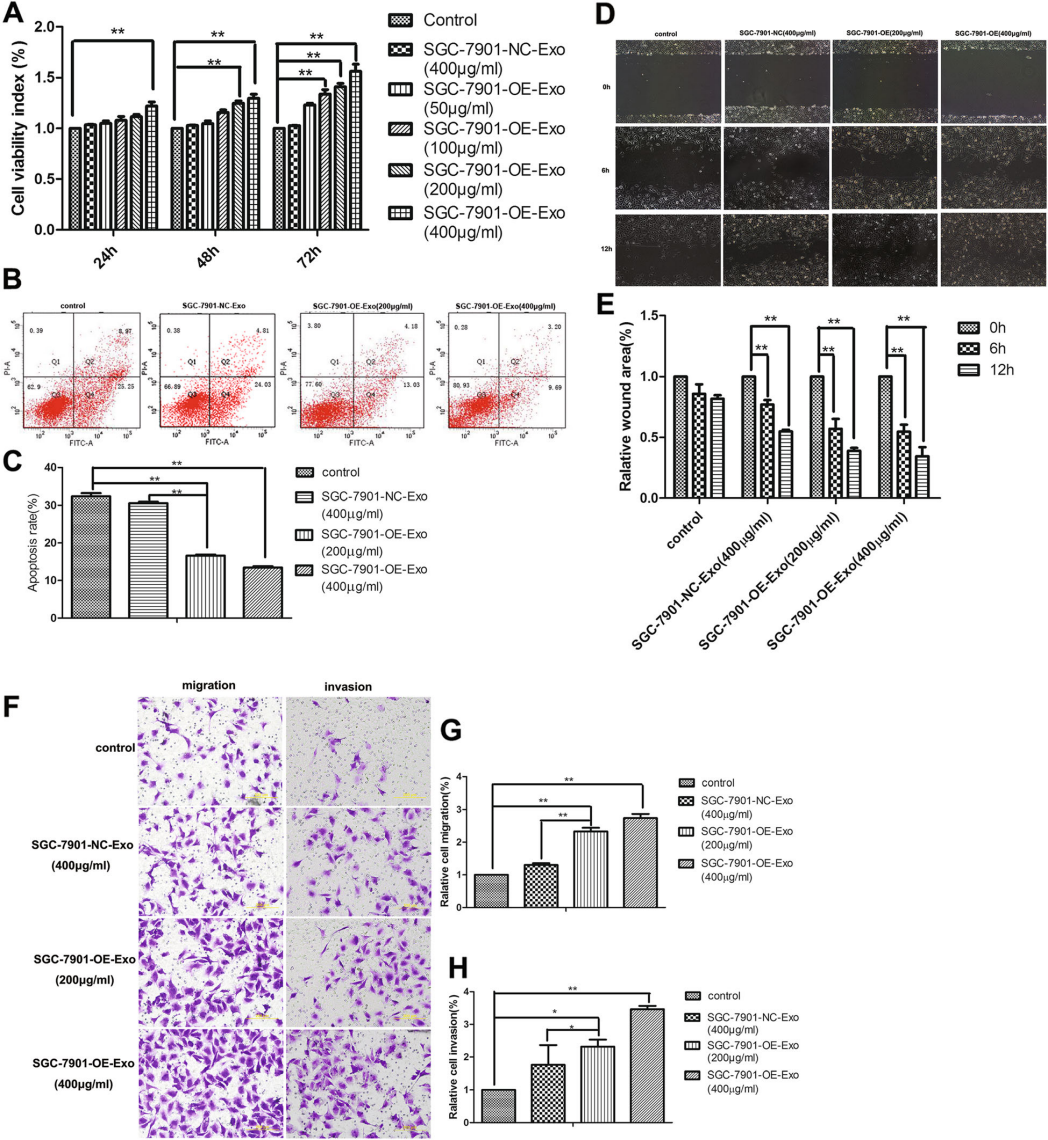
The influence of exosomes from YB-1-upregulated GC cells on the proliferation, apoptosis, migration and invasion of HUVECs. **a** MTT assays revealed that SGC-7901-OE-Exo promoted the proliferation of HUVECs. **b** Apoptosis assays using flow cytometry and (**c**) the corresponding quantitative analysis demonstrated that SGC-7901-OE-Exo reduced the apoptosis of HUVECs. **d** Scratch wound healing assays for HUVECs in different conditions. **e** The level of cell migration into the wound scratch was quantified as the wound healing area and compared to that of control group at 6 and 12 h. **f** The transwell pictures and (**g**, **h**) the quantitative data revealed significantly increased migration and invasion of HUVECs treated by SGC-7901-OE-Exo than the control group. (*, *P* < 0.05; **, *P* < 0.01)

Based on MTT assays, SGC-7901-NC-Exo at 400 μg/ml slightly increased the proliferation of HUVECs comparing with PBS, but no statistical significance was shown. Whereas comparing with PBS and SGC-7901-NC-Exo, SGC-7901-OE-Exo significantly promoted the proliferation of HUVECs in a dose-and time-dependent manner (*p* < 0.01), especially at 400 μg/ml for 72 h (Fig. 3a).

As shown in Fig. 3b and c, the apoptosis of HUVECs was significantly decreased by 200 and 400 μg/ml SGC-7901-OE-Exo, but not SGC-7901-NC-Exo or PBS, and the apoptotic rate was 16.62 ± 0.52%, 13.45 ± 0.56%, 18.59 ± 0.72% and 32.33 ± 1.44%, respectively (*p* < 0.01).

### Exosomes from YB-1-upregulated GC cells accelerate migration of HUVECs

Comparing with SGC-7901-NC-Exo or PBS, SGC-7901-OE-Exo greatly strengthen the migration ability of HUVECs in a time and dose dependent manner (Fig. 3d, e; *p* < 0.01). Either higher concentration of SGC-7901-OE-Exo (400 μg/ml) or longer incubation time (12 h) could result in more obvious decrease of wound area of HUVECs, in comparison with the lower concentration of SGC-7901-OE-Exo (200 μg/ml) or shorter incubation time (6 h). Nontheless, 400 μg/ml SGC-7901-NC-Exo was demonstrated to slightly (but not significantly) enhance the migration property of HUVECs and the speed of scratch wound healing in comparison with PBS.

### Enhanced migration and invasion of HUVECs observed in transwell assays

With transwell assays, it was further confirmed that SGC-7901-OE-Exo promoted the migration ability of HUVECs in a concentration dependent manner, based on the observations that 400 μg/ml SGC-7901-OE-Exo induced more migrated cells than 200 μg/ml SGC-7901-OE-Exo, SGC-7901-NC-Exo and PBS. Consistently, the number of invaded HUVECs was observed significantly higher after the treatment of SGC-7901-OE-Exo than the treatment of SGC-7901-NC-Exo or PBS (*P* = 0.007 and 0.0002). Furthermore, the invasion efficacy of HUVECs triggered by SGC-7901-OE-Exo was also concentration dependent, and that SGC-7901-OE-Exo of 400 μg/ml promoted the invasion of more HUVECs than that of 200 μg/ml (Fig. 3f, g, h; *P* = 0.013 and 0.003).

### Exosomes from YB-1-upregulated GC cells facilitate in-vitro angiogenesis

**Fig. 4:**
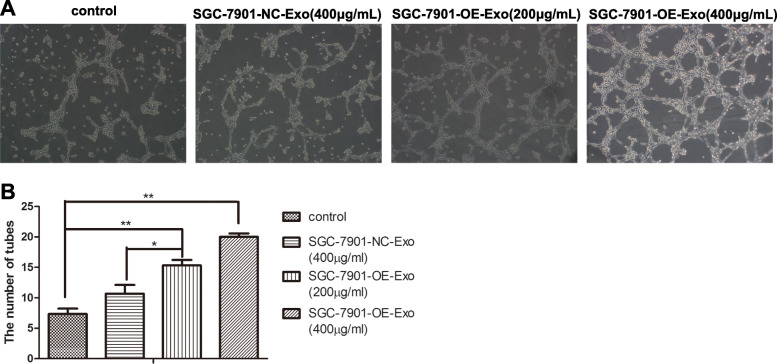
Exosomes from YB-1-upregulated GC cells facilitate in-vitro angiogenesis. **a** Photographs of tube formation was taken at 12 h to show that the HUVECs treated by SGC-7901-OE-Exo formed significantly greater number of tubes on matrigel than controls. **b** Quantitation of tube formation was shown. Values represent averages ±SE of three independent measurements along the wound scratch. (*, *P* < 0.05; **, *P* < 0.01)

As shown in Fig. 4, SGC- 7901-OE-Exo significantly increased the neovasculature property of HUVECs, thus promoted more tube formations comparing with SGC- 7901-NC-Exo and PBS (*p* < 0.01). Furthermore, there was more formation of microtubes when HUVECs were incubated with higher concentration of SGC- 7901-OE-Exo (400 μg/ml) than lower concentration (200 μg/ml).

### Quantity assays for VEGF, Ang-1, MMP-9 and IL-8 in HUVECs and the treating exosomes

**Fig. 5:**
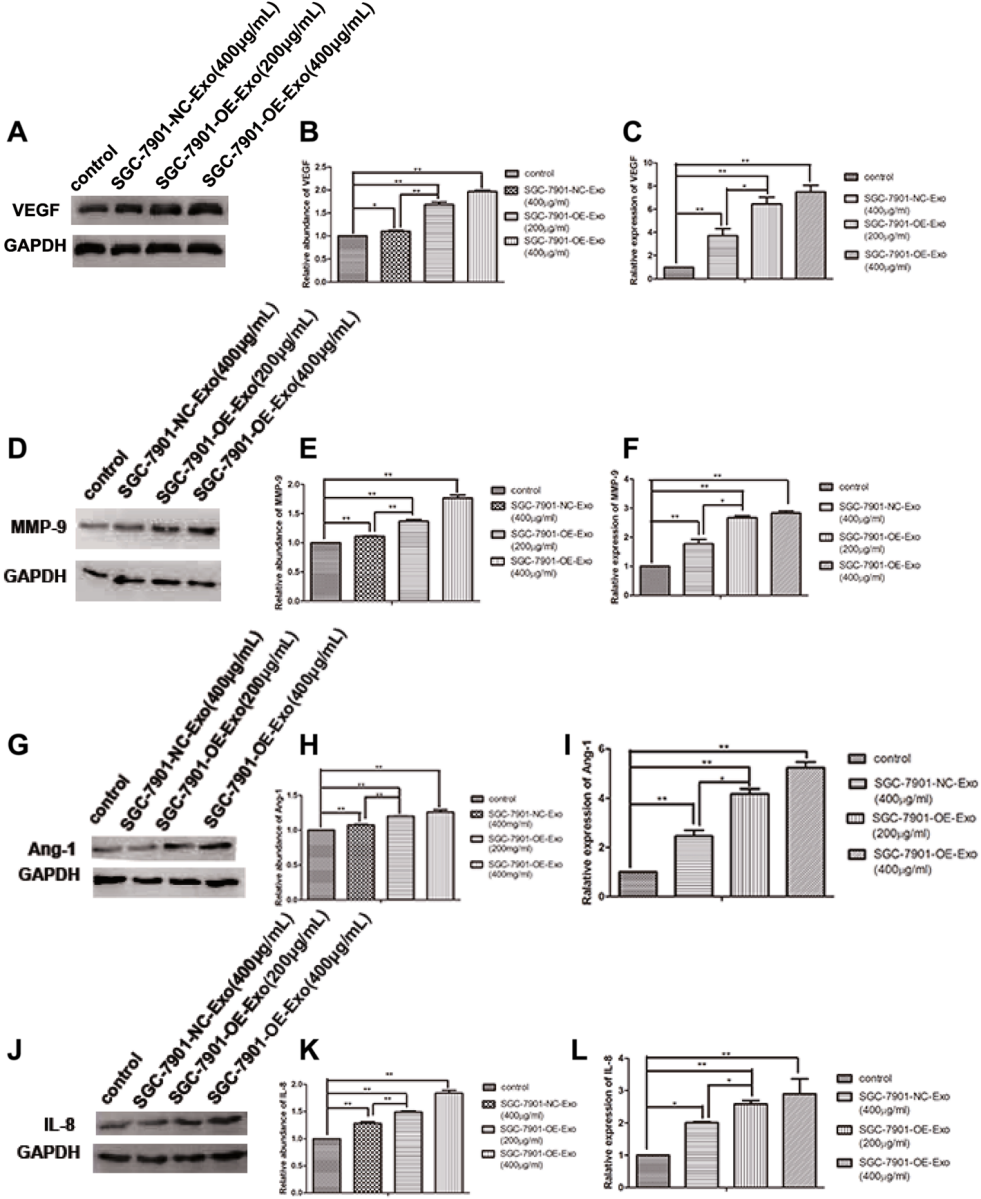
Quantity assays for VEGF, MMP-9, Ang-1 and IL-8 in HUVECs. VEGF expression was analyzed using western blot (**a**) and the corresponding quantitative analysis (**b**), and qRT-PCR assays (**c**) in HUVECs stimulated with exosomes. MMP-9 was identified by western blot (**d**) and the corresponding quantitative analysis (**e**), and qRT-PCR (**f**) in HUVECs stimulated with exosomes. Ang-1 expression was analyzed using western blot (**g**) and the corresponding quantitative analysis (**h**), and qRT-PCR assays (**i**) in HUVECs stimulated with exosomes. IL-8 was identified by western blot (**j**) and the corresponding quantitative analysis (**k**), and qRT-PCR (**l**) in HUVECs stimulated with exosomes. (*, *P* < 0.05; **, *P* < 0.01)

In HUVECs, the protein levels of angiogenetic factors including VEGF, Ang-1, MMP-9 and IL-8 were found to be upregulated by SGC-7901-NC-Exo and SGC-7901-OE-Exo, but not PBS. Given that the four angiogenetic factors showed gradual increase in the HUVECs, respectively, treated by 400 μg/ml SGC-7901-NC-Exo, 200 μg/ml and 400 μg/ml SGC-7901-OE-Exo, the expressions of the four angiogenetic factors are supposed to be correlated with the amount of exosome-transferred YB-1 to HUVECs (Fig. 5a, b, d, e, g, h, j, k). Consistent with the western-blot results, qRT-PCR confirmed the mRNA upregulation of the four factors. Comparing with PBS treatment, the increase of mRNA in HUVECs was, respectively, shown to be 2.71, 5.45 and 6.49 folds for VEGF; 1.45, 3.18 and 4.23 folds for Ang-1; 0.77, 1.67 and 1.83 folds for MMP-9; and 0.99, 1.58 and 1.89 folds for IL-8; following the treatment of 400 and 200μg/ml SGC-7901-OE-Exo and 400 μg/ml SGC-7901-NC-Exo (Fig. 5c, f, i, l).

Based on RNA sequencing analysis for the exosome mRNAs, we failed to demonstrate the statistically increase of the mRNA of VEGF, Ang-1, MMP-9 and IL-8 in the SGC-7901-OE-Exo comparing with SGC-7901-NC-Exo, and the | log 2(fold change) | was, respectively, shown to be 0.41, 0.26, 1.21, and 0.34. However, SGC-7901-OE-Exo transferred significantly higher YB-1 mRNA than SGC-7901-NC-Exo between GC and HUVECs, as demonstrated by the | log 2(fold change) | of 1.78.

## Discussion

Tumor-derived exosomes are transmission vesicles mediating intercellular communications and participating in multiple cancer procedures, such as tumor microenvironment remodeling, immune response, angiogenesis, invasion, metastasis and drug-resistance [8]. Although GC-derived exosomes have been largely studied since the first characterization decades ago [20], the publications on the regulation of GC angiogenesis are limited. So far, a few of cargoes including CD44, CCR6, HER-2/neu [21] and several cytokines [22] were identified in GC-derived exosomes, and miR-130a/C-MYB [23], miR-135b/FOXO1 [24], miR-155/FOXO3 [25] and HGF [26] were, respectively, uncovered to be potential signals for exosome-HUVEC interactions. Given that the complex compositions including receptors, transcription factors, enzymes, extracellular matrix proteins, lipids, nucleic acids (DNA, mRNA, and miRNA) constitute exosomes, far further declarations on the complex working mechanisms of exosomes in GC angiogenesis are needed. Herein, we showed that exosomes purified from GC cell lines contain the multiple functional factors YB-1, and exosomal YB-1 upregulation could boost the migration and angiogenesis of HUVECs. Interestingly, both mRNA and protein level of angiogenic factors including IL-8, VEGF, Ang-1, and MMP-9 in HUVECs were disclosed to be elevated, whereas their mRNA in cancer-derived exosomes showed no difference between YB-1 upregulated and the control cells.

Accordingly, YB-1 overexpression predicted poor prognosis of patients in multiple cancers including GC [27], and the upregulation of YB-1 in either GC cells or cancer vascular endothelial cells, respectively, indicated advanced cancer stage and more active angiogenesis based on the analysis of GC specimens [17, 18]. Moreover, in-vitro studies have focused on the role of YB-1 on drug resistance and cell cycle regulation in GC, while there is still no publication on the mechanisms of YB-1 facilitating angiogenesis. In the current study, we successfully detected YB-1 in GC-derived exosomes, and found that the expression level of YB-1 in GC cells and the corresponding purified exosomes were consistent among various GC cell lines. Exosomes derived from GC cells were demonstrated to be taken up by HUVECs. By the co-incubation of GC derived exosomes with HUVECs, it was observed that the exosomes derived from SGC-7901 LV-YBX1 cells could concentration dependently promote the proliferation, migration, invasion, angiogenic tube formation, and inhibit the apoptosis of HUVECs comparing with those from SGC-7901 LV-NC cells. As far as we know, this is the first study to declare that YB-1 is transferred from GC cells into endothelial cells to promote cancer angiogenesis through vesicle transportation.

To explore the further mechanisms for YB-1 mediated crosstalk between GC and endothelial cells, we detected the angiogenic factors in HUVECs treated by two types of exosomes and compare their mRNA levels in the corresponding exosomes. Notably, we observed that both mRNA and protein levels of IL-8, VEGF, Ang-1 and MMP-9 in HUVECs were obviously increased upon the treatment of exosomes derived from SGC-7901 LV-YBX1 cells, whereas mRNAs of the four angiogenic factors were similar in the two types of exosomes.

It has been identified that IL-8, VEGF, MMP-9, and Ang-1 are all important angiogenic factors upregulated in HUVECs induced by various irritators [28–30]. Numerous studies implicate that YB-1 involves in VEGF pathways, and anti-YB-1 treatment mediates decreased VEGF signals and tumor regression [31]. In addition, the only report about YB-1 mediated IL-8 regulation is the study for senescence of keratinocyte progenitors, and found that YB-1 stabilizes IL-8 mRNA by binding its 3′-untranslated regions (3’UTR) and prohibit IL-8 expression [14]. As for MMP-9 and Ang-1, there is still no report about the relationship with YB-1 so far. We currently revealed the role of YB-1 for the upregulation of VEGF, IL-8, MMP-9, and Ang-1 in HUVECs during the exosome-mediated-angiogenesis in GC, with detailed mechanisms deserving further illustrations.

According to the previous publications, cancer-derived exosomes initiate or suppress signaling pathways in the recipient cells via transmitting various cargoes. Among the cargoes, mRNAs stabilized in the form of mRNPs are important [32]. YB-1 is regarded as the key composition to stabilize mRNAs in mRNPs [32], and one of the mechanisms is that YB-1 bind 3’UTR of mRNAs to stabilize mRNAs and modulate their translation in cell cytosol [12, 33]. In our study, mRNA of the four angiogenic factors all demonstrate the similar level in the two types of exosomes (YB-1 upregulated or not). Therefore, the upregulation of the four angiogenic factors might not be caused by the mRNA-stabilization function of YB-1 in the exosomal mRNPs.

**Fig. 6:**
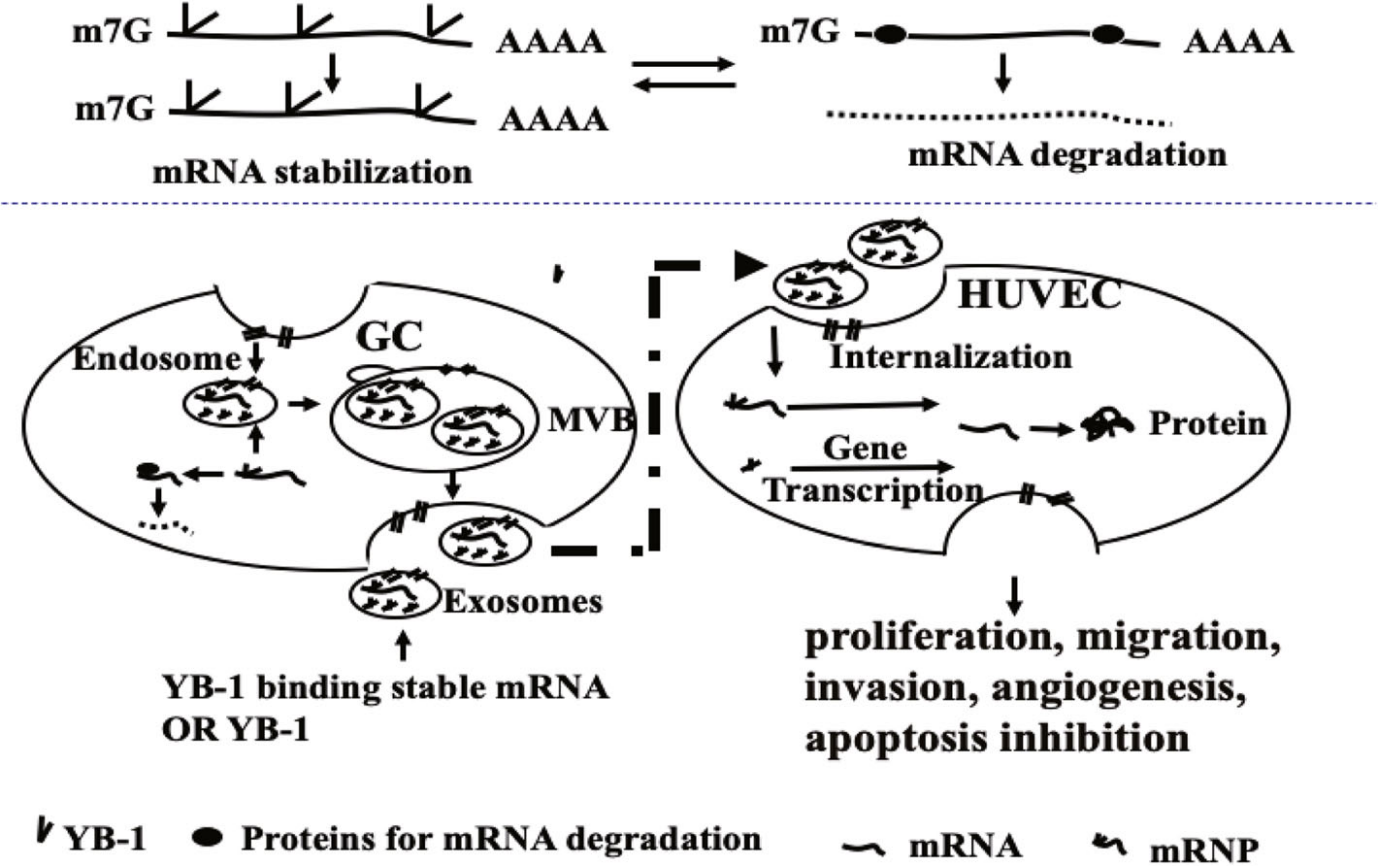
The supposed mechanisms of YB-1 promoting angiogenesis in gastric cancer

As is known, YB-1 not only involves in protein translation by modulating mRNA localization and stability [13, 14], but also mediates transcriptional control as a well-characterized transcription factor with the ability to translocate from the cytosol to the nucleus [13]. Therefore, we hypothesis that the elevated expression of the four angiogenic factors in HUVECs might due to the enhanced transcription mediated directly by more YB-1 transferred via exosomes, or indirectly by more other angiogenic upstream factors with their mRNA stabilized by YB-1 and the supposed mechanism of YB-1 promoting angiogenesis in GC was shown in Fig. 6.

Given that there are much more pro-angiogenic related factors like VEGFR, Ang2/Tie2, and IL6 might be related with GC angiogenesis, further work should be done to more clearly clarify the molecular mechanisms of YB-1 mediated GC angiogenesis via exosomes.

## Conclusion

Overall, our study reveals that YB-1 is involved in the genetic communication between GC cells and HUVECs via exosomes, and plays a role in the neovasculature and premetastatic niche formation induced by cancer exosomes in GC. Further research on the working mechanisms of YB-1 in exosomes for GC angiogenesis are warranted, and more clinical studies could be done to reveal whether YB-1 can be a potential biomarker for predicting invasion or metastasis in GC.

## Data Availability

The datasets supporting the conclusions of this article are included within the article. Any request of data and material may be sent to the corresponding author.

## Abbreviations

GC: Gastric cancer
YB-1: Y-box binding protein 1
HUVECs: Human umbilical vein endothelial cells
VEGF: Vascular endothelial growth factor
Ang-1: Angiopoietin-1
MMP-9: Matrix metalloprotein-9
IL-8: Interleukin-8
PCR: Polymerase chain reaction
Exo: Exosome
DAPI: 4′,6-diamidino-2-phenylindole
DMSO: Dimethyl sulfoxide
OD: Optical density
FITC: Fluorescein Isothiocyanate
PI: Propidium iodide
FBS: Fetal bovine serum
RT-PCR: Reverse Transcription-Polymerase Chain Reaction
PBS: Phosphate buffer saline
SD: Standard deviation
PVDF: Polyvinylidene difluoride
GAPDH: Glyceraldehyde-3-phosphate dehydrogenase
